# Application of Physiologically-Based Pharmacokinetic (PBPK) Model in Drug Development and in Dietary Phytochemicals

**DOI:** 10.1007/s40495-025-00427-w

**Published:** 2025-08-08

**Authors:** PoChung Chou, Ahmad Shannar, Yuxin Pan, Parv Dushyant Dave, Jiawei Xu, Ah-Ng Tony Kong

**Affiliations:** 1 Graduate Program in Pharmaceutical Science, Ernest Mario School of Pharmacy, Rutgers, The State University of New Jersey, Piscataway, NJ 08854 USA; 2 Department of Pharmaceutics, Ernest Mario School of Pharmacy, Rutgers, The State University of New Jersey, Piscataway, NJ 08854 USA

**Keywords:** PBPK, Lead optimization and candidate evaluation, Drug-drug interactions, Formulation simulation, Extension to special population, Dietary phytochemicals

## Abstract

**Purpose of review:**

Physiologically-based pharmacokinetic (PBPK) modeling is a powerful tool to understand drug movements throughout the human body. Unlike classical PK methods that often lack sufficient physiological detail, PBPK integrates drug-specific properties with organism-specific physiological parameters to predict drug behavior in major body compartments, particularly site of action and providing high physiological realism. The aim of the review is to summarize application of PBPK modeling in drug development and in dietary phytochemicals.

**Recent findings:**

PBPK modeling is a versatile tool in drug development and phytochemical research. It predicts human PK from preclinical data, aiding lead optimization and candidate evaluation. The model mechanistically predicts drug-drug interactions (DDIs), supporting dose adjustments and reducing clinical trials. PBPK also enables formulation simulation for oral and modified-release drugs, optimizing bioavailability and predicting performance from in vitro data, thus reducing costly in vivo studies. Importantly, it extends drug knowledge to pediatric and special populations via virtual group simulations, enabling efficient, cost-effective dosage determination and less clinical trials. For dietary phytochemicals, PBPK modeling is well-suited for their complex mixture and variability. PBPK studies of phytochemicals demonstrate their utility for single components, mixtures, cross-species extrapolation, and complex metabolic processes, although challenges exist.

**Summary:**

PBPK modeling is a dynamic and quantitative tool offering comprehensive pharmacokinetic integration across various populations and regimens. Its importance is growing due to its application at diverse stages of drug development and its ability to adapt to complex substances, including natural products. Ultimately, PBPK modeling is significant for enhancing scientific rigor, expediting drug development and ensuring patient safety.

**Graphical Abstract:**

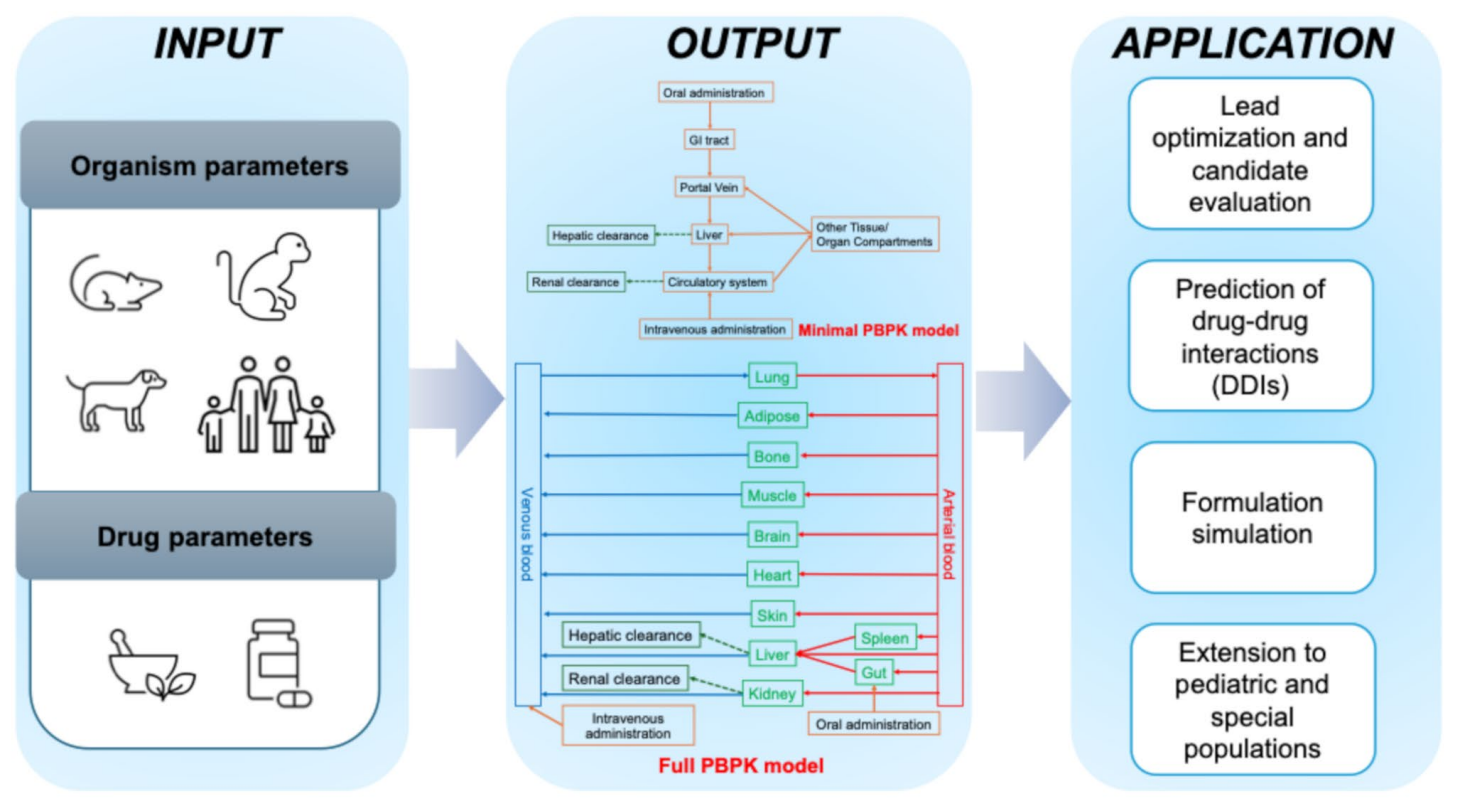

## Introduction

Pharmacokinetic (PK)/pharmacodynamic (PD) modeling elucidates the drug concentration profile in the blood, at different tissues, and most importantly at the site of action over time, integrated with drug efficacy and or toxicity, following drug administration into the body [1, 2]. With the advances of various PK/PD software, leveraging these PK/PD software platforms, such as Phoenix WinNonlin, NONMEM, GastroPlus, Simcyp, and the Monolix suite, the absorption, distribution, metabolism, and excretion (ADME) of a drug in the body are comprehensively analyzed. Subsequently, the PK parameters responsible for the observed profile, including C_max_ (maximum plasma drug concentration), T_max_ (time to reach maximum plasma concentration (C_max_) in the bloodstream following drug administration), V_d_ (apparent volume of distribution), AUC (area under the plasma concentration-time curve), CL (apparent total body clearance of the drug from plasma), and T_1/2_ (elimination half-life of the drug), will be derived. In addition, in conjunction with the PD analysis, which typically describes the effect/response elicited by the drug at the site of action, the PK/PD analysis enables researchers to understand the characteristics of drug exposure, determine an appropriate dose for a clinical study, assess changes in dose requirements, and establish safety margins and efficacy characteristics during drug development [1, 3].

The classical PK compartmental analysis characterizes the drug’s ADME profiles in the central and peripheral compartments, which is frequently insufficient to delineate the complete PK properties in the representative tissues and organs. However, physiologically-based pharmacokinetic (PBPK) modeling is a powerful and advanced approach that is accepted by the US Food and Drug Administration (FDA) for new drug application and regulatory decision with the incorporation of the physiological parameters of human or other species coupled with the physicochemical and biological properties of the drug to predict and estimate the PK profiles of the drug in the body’s major compartments, such as the blood, liver, kidney, and the gastrointestinal (GI) tract [4, 5]. In contrast to traditional PK modeling, which employs a “top-down” approach by utilizing extensive experimental data to characterize the PK properties of the drug, the PBPK modeling typically adopts a “bottom-up” methodology to simulate the PK profiles of the drug within the major physiological compartments [4, 6, 7]. The prediction of the bottom-up approach, nevertheless, is not always fitted to the observed data. As a result, a “middle-out” approach, which integrates “bottom-up” and “top-down” methodologies, is frequently employed in PBPK analysis to parameterize models due to scientific knowledge gaps [8].

In general, PBPK workflow, three types of parameters are considered and collected from in vitro-in vivo extrapolation (IVIVE) approaches and clinical data. Organism parameters, including system parameters, which are species- and population-specific due to physiological properties variation among different animal species and special populations (e.g., healthy versus diseased populations). These parameters include organ volumes, organ compositions, blood flow, and surface areas. Drug parameters, also known as the physicochemical properties of the drug, provide the drug’s fundamental characteristics, including compound lipophilicity (log^P^, log^D^), solubility, molecular weight (MW), and pK_a_ values, which are independent of organisms. The interaction between the organism’s biological system and the drug parameterizes drug-biological properties, such as the fraction of the drug unbound (f_u_) and tissue-plasma partition coefficient (K_p_) [9, 10]. In addition to the fundamental parameters, other parameters, such as metabolic enzymes and transporters, aid in enhancing the fitness of the model.

Dietary phytochemicals are found abundantly in fruits, vegetables, grains, medicinal herbs, and other natural sources; hence, dietary phytochemicals, in general manifest lower toxicity when consumed in the longer term [11, 12]. Increasing research has demonstrated that dietary phytochemicals possess antioxidant, anti-inflammatory, antiangiogenic abilities, and anti-cancer effects, including epigenetic modifications based on their natural structures [13, 14]. Promising preclinical evidence has been unveiled; however, a significant limitation to the clinical application of dietary phytochemicals including their low oral bioavailability due to gut metabolism, among others [15, 16]. For instance, resveratrol exhibits poor oral bioavailability in PK studies, due to its rapid and extensive liver metabolism [17]. To augment the bioavailability of dietary phytochemicals, several strategies are being explored, including the combination with other drugs or phytochemicals [17], as well as the utilization of advanced drug delivery formulations [18, 19], such as nanoparticles. But most importantly, due to the nature of the complex mixture of dietary phytochemicals found in fruits, vegetables, grain, medicinal herbs, and other natural sources, limited information is available in humans for each of the bioactive ingredient’s PK and PD profiles including ADME, tissue distribution, dose-response, among others. Since PBPK, which is a powerful modeling tool to predict the potential drug concentration in humans in hard to access tissue/organ sites such as the brain and the heart, drug-drug interactions (DDI), impact of diseases, age, genetics/epigenetics, among others coupled with the advent of PK/PD software, PBPK modeling could offer a comprehensive integration of drug absorption, metabolism, and transport processes of dietary phytochemicals. It could effectively simulate the PK and PD profiles for each of the bioactive phytochemicals, variations in drug exposures and interactions across diverse populations with varying dosage regimens. The PBPK methodology not only enhances the scientific rigor of early bioactive phytochemical screening and clinical trial design but also provides a robust tool to mitigate potential safety concerns in clinical trials and expedite the development of novel bioactive dietary phytochemicals. As such, this PBPK modeling approach is particularly well-suited for natural products, characterized by intricate material composition and limited clinical data. The application of PBPK on dietary phytochemicals will be further discussed below in the section on PBPK Modeling of Dietary Phytochemicals.

### Building Blocks of a PBPK Model

PBPK modeling is a mechanistic approach that quantitatively describes the ADME of compounds by integrating drug-specific properties with organism-specific physiological parameters. It is widely employed during drug development to predict human PK, optimize clinical trial designs, and assess drug safety and efficacy in special populations. In the following sections, we will discuss three topics: (I) construction of a PBPK model and a concise overview of common PBPK software; (II) PBPK model assumptions and mathematical equations; (III) comparison of a full PBPK model and a minimal PBPK model.

#### Construction of a PBPK Model and a Concise Overview of Common PBPK Software

The construction of a PBPK model comprises five distinct phases: defining the model architecture, gathering system-specific data, integrating compound-specific data, calibrating and validating the model, and applying it for human prediction and simulation purposes.

The initial phase entails defining the anatomical compartments that represent diverse tissues and organs. A full PBPK model typically incorporates physiological compartments such as the liver, kidneys, gut, brain, lungs, heart, adipose tissue, muscle, and blood, among others. Each compartment is characterized by organ-specific volumes, blood flows, and partition coefficients. Subsequently, species-specific anatomical and physiological parameters (e.g., tissue volumes, blood flow rates, plasma protein levels) are incorporated. Standardized databases and literature sources frequently provide these parameters for humans and common preclinical species. Drug-related parameters, including molecular weight, Log^P^, pK_a_, permeability, plasma protein binding, metabolic clearance rates, and transporter interactions, are also integrated. These parameters are typically obtained from in vitro assays or computational predictions. Following the input of model-required parameters and the acquisition of a preliminary PBPK model, the model undergoes calibration using available in vivo PK data and is subsequently adjusted as necessary to enhance its predictive performance. Validation is then conducted employing independent datasets that are not utilized during model development. Finally, a validated PBPK model can simulate concentration-time profiles in plasma and tissues under various dosing regimens, predict DDIs, assess the impact of disease or genetics, and support regulatory submissions.

Numerous professional PBPK software platforms are extensively utilized in academic and industrial settings. We summarize the common PBPK software in Table 1, which is categorized by software, developer, key features, typical applications, and access type. For instance, Simcyp, developed by Certara, is widely employed for predicting DDIs, pediatric PK, and regulatory submissions. Gastroplus, built by Simulation Plus, specializes in modeling oral absorption and dissolution, integrating physiology-based biopharmaceutics modeling. PK-Sim from Open Systems Pharmacology is an open-source platform that offers capabilities for whole-body PBPK modeling across species. Each platform provides a comprehensive suite of built-in libraries, parameter estimation tools, and simulation modules, meticulously tailored to address various stages of drug development.

**Table 1:** Overview of commonly used PBPK modeling software platforms

Software	Developer	Key Features	Typical Applications	Access Type
Simcyp^®^ Simulator	Certara	• Extensive physiological libraries • DDI prediction, pediatric modeling • Special population simulations • Virtual population modeling • Disease progression modeling • Physiologically-based scaling of drug absorption and distribution • Advanced simulations for biotechnology products	• Human PK prediction • DDI assessment • Pediatric and special population modeling • Regulatory submissions • Drug development • Disease progression modeling • Virtual clinical trials • Clinical trial simulation for biologics	Commercial (licensed)
PK-Sim^®^	Open Systems Pharmacology	• Whole-body PBPK modeling • Cross-species extrapolation • Open-source community support	• Human and animal PK modeling • Early discovery to development	Open-source (free)
GastroPlus^®^	Simulations Plus	• Advanced absorption and dissolution modeling • Gut physiology simulation, formulation optimization • PBPK for oral and IV dosing • IVIVC (in vitro-in vivo correlation), • Gastric emptying and drug release profiles • DDI simulations • Physiologically-based scaling for dose predictions • Simulation of food effects on absorption	• Oral absorption modeling, formulation optimization • FIH dose prediction, • DDI prediction • Simulation of food effects on drug absorption • Optimization of drug release profiles • Oral dose and bioavailability prediction	Commercial (licensed)
Berkeley Madonna^®^	UC Berkeley	• General-purpose differential equation solver • Customizable models	• Custom PBPK and QSP model development • Academic purpose	Commercial (licensed)
MoBi^®^	Open Systems Pharmacology	• Extension of PK-Sim^Ⓡ^ for detailed intracellular processes and systems biology	• Systems pharmacology • Intracellular PK modeling	Open-source (linked to PK-Sim^®^)
MATLAB^®^ SimBiology^®^	MathWorks	• Flexible modeling environment • Customizable PBPK and Quantitative systems pharmacology (QSP) models	• Research purpose • Systems pharmacology, academic PBPK modeling	Commercial (licensed)

*The information is excerpted from the official website of each company

#### Two PBPK Model Assumptions and Mathematical Equations

Drug distribution and transport from blood to tissue compartments are mainly delineated by two assumptions: perfusion-limited (flow-limited) and permeability-limited (diffusion-limited) assumptions [20]. Drug movement between different tissues is simulated using differential equations based on mass balance principles for each compartment. Each compartment can be described as a simple static compartment with passive permeability or a more mechanism-based compartment that incorporates variables such as transporters, metabolic enzymes, and distinctive drug permeability [21]. Two major assumptions that define the type of model structure are presented below:

#### Perfusion-Limited (Flow-Limited) Model

The perfusion-limited model is typically applied to small lipophilic substances. The transport of a drug from blood to tissue is dependent on blood flow rates; consequently, the entry of the drug into the tissue compartment is restricted by blood perfusion [22]. This model assumes that drug permeation across tissue membranes is rapid compared to blood flow, making blood flow the rate-limiting step. Perfusion-limited models can be categorized into two types: one based on the assumption of a well-stirred compartment, where there is no concentration gradient, and the other derived from a dispersion model, where concentration gradients exist despite the absence of an identifiable diffusion barrier [23]. Tissue uptake in perfusion-limited models for a tissue compartment can be expressed in Eq. (1):

1 $$\:\frac{d{C}_{i}}{dt}=\:\frac{{Q}_{i}}{{V}_{i}}\:(\:{C}_{blood}-\:\frac{{C}_{i}}{{K}_{p,i}}\:)$$

where C_i_ is the tissue concentration, Q_i_ is the blood flow rate to the tissue, V_i_ is the compartment volume of each tissue, K_p, i_ is the tissue-to-plasma partition coefficient, and C_blood_ is the arterial blood concentration [20]. The K_p, i_ can be estimated from in vitro or in vivo distribution data (ratio of tissue and plasma concentrations at steady-state) or can be obtained using quantitative structure/property relationship (QSPR) modeling [20].

#### Permeability-Limited (Diffusion-Limited) Model

The permeability-limited model, on the other hand, is generally utilized for larger polar molecules. The drug distribution to tissue compartments is limited by the permeability of the cellular membrane. In the permeability-limited model, each tissue is partitioned into two compartments: the intracellular space and the extracellular space, which are separated by a cell membrane barrier and could be described separately [24]. The concentration of drugs will attain equilibrium in permeability-limited models; however, the time to achieve equilibrium is particularly dependent on the substance-specific permeability instead of blood flow [25]. Equations (2) and (3) incorporate the permeability of the tissue and describe tissue uptake in the vascular compartment and the extravascular compartment, respectively:

- Vascular compartment:

2 $$\:\frac{d{C}_{i,\:v}}{dt}=\:\frac{{Q}_{i}}{{V}_{i,\:v}}\:\left(\:{C}_{blood}-\:{C}_{i,\:v}\right)-\frac{{P}_{i}}{{V}_{i,v}}\:\left(\:{C}_{i,\:v}-\:\frac{{C}_{i,\:ev}}{{K}_{p,i}}\right)$$

- Extravascular compartment:

3 $$\:\frac{d{C}_{i,\:ev}}{dt}=\:\frac{{P}_{i}}{{V}_{i},ev}\:\left(\:{C}_{i,v}-\:\frac{{C}_{i,\:ev}}{{K}_{p,i}}\right)$$

where C_i, v_ and C_i, ev_ represent drug concentrations in the vascular and extravascular compartments, P_i_ is the permeability of the tissue. V_i, v_ and V_i, ev_ are the compartment volumes of the vascular and extravascular compartments, respectively [20, 26].

To enhance the predictive power of PBPK models, both frameworks can integrate additional parameters pertaining to enzymatic metabolism, transporter utilization, and active uptake mechanisms, among others.

#### Comparison of a Full PBPK Model and a Minimal PBPK Model

The adoption of a PBPK model is contingent upon the specific application, objectives, data availability, and regulatory expectations. Consequently, the model can be complex (full PBPK model) or simple (minimal PBPK model) [21].

A full PBPK model, referred to as a whole-body PBPK model, encompasses a detailed set of tissue compartments, including the liver, kidneys, gastrointestinal tract, brain, lungs, heart, adipose tissue, and muscles, all interconnected with the circulatory system. Each compartment is characterized by its organ-specific blood flow, tissue volumes, and intricate mechanistic processes. A full PBPK model provides high physiological realism and holds significant value in predicting tissue concentrations, assessing DDIs, and simulating the kinetics of specialized populations. However, it demands substantial input data and incurs substantial computational costs [4].

In contrast, a minimal PBPK model simplifies the entire body structure by lumping tissues with comparable kinetics into a single compartment to reduce complexity. In Cao and Jusko’s research article, minimal PBPK models with one, two, and three tissue compartments were examined, and the pertinent mass balance differential equations were elucidated [6]. Although minimal PBPK models offer less granular information, they can still capture essential PK characteristics and are beneficial when input data is constrained or rapid simulations are necessitated.

### Application of PBPK modeling in Drug Development

PBPK modeling is extensively employed in drug development, which is typically divided into three phases: discovery, early development, and late development [9, 27]. It plays pivotal roles in each phase, including providing valuable insights into drug exploration, facilitating clinical development decisions, and promoting regulatory communication [28]. During the initial discovery phase, the application of high-throughput screening technology and PBPK modeling provides valuable information about lead molecule identification and optimization, as well as candidate evaluation, thereby facilitating decision-making and the development strategy. As the development progresses, PBPK modeling becomes increasingly involved in predicting human PK, including the first-in-human (FIH) dose and DDIs. It also assists in formulation development and developing a strategy for pediatric and special populations [27]. We will discuss the four major applications of PBPK modeling below.

#### Lead Optimization and Candidate Evaluation

In the early stages of drug discovery, when insufficient data are available to accurately formulate PBPK models, PBPK modeling facilitates rational lead optimization by predicting the human PK profiles of candidate compounds based on the utilization of physicochemical properties, in vitro data, and in silico data [28]. In the stage of lead optimization, the PBPK model plays a crucial role in facilitating human PK and exposure prediction, as well as the selection of the FIH dose. For instance, prior to the prediction of human PK for a lead compound, a preliminary PBPK model is constructed by incorporating system and drug parameters derived from in vitro data. Subsequently, the model undergoes modifications and validation by in vivo PK profiles. Once the preclinical PBPK model has been validated, the same drug parameters, combined with human-specific in vitro data pertaining to clearance, plasma protein, and microsomal binding, are further employed to simulate human PK profiles for various administration regimens, including the FIH prediction [27, 28].

Furthermore, PBPK modeling in lead optimization boosts the identification of ADME liabilities and supports medicinal chemistry endeavors. Potential challenges, such as compromised permeability, metabolism, or extensive first-pass extraction, can be identified during the early stage, enabling the prioritization of compounds exhibiting favorable PK characteristics. These insights can be utilized to optimize the candidate structure toward enhanced absorption, distribution, metabolic stability, elimination profiles, toxicity, and safety evaluation [29, 30]. Overall, PBPK modeling during lead optimization expedites decision-making, diminishes clinical attrition rates, and augments the likelihood of successful candidate drug development.

#### Drug-drug Interaction (DDI) Potential Prediction & Human PK and DDI Prediction To Prevent Clinical DDI Trials

Drug-drug interaction (DDI) is a phenomenon in which the efficacy of one or more drugs is altered due to the interaction of two or more drugs in the body during simultaneous or sequential use [31]. This interaction may enhance the efficacy of the drug, or it may weaken or even cause toxicity. With the increasing need for combination therapy for multiple diseases, the chances of DDIs have risen significantly. Studies have shown that more than 195,000 people are hospitalized annually in the United States due to inappropriate use of medications, and more than two million people are hospitalized annually in China due to drug-to-drug adverse reactions, indicating that DDIs have become a global public health concern [32].

The significance of DDIs research lies not only in preventing potential harm but also in optimizing drug combination therapy strategies to improve the safety and efficacy of treatment. By identifying and predicting DDI risks at an early stage, a more rational dosing regimen can be formulated at the drug development phase, avoiding the risk of drug withdrawal due to severe DDIs after marketing [33]. The development and establishment of modern modeling tools, such as PBPK modeling and databases, have significantly enhanced the scientific and systematic approach to predicting and managing DDIs. Consequently, an in-depth study of DDIs has emerged as a crucial component in ensuring patient safety and optimizing the efficacy of drug therapies.

By integrating drug properties, in vitro metabolism/transport data, and human physiological parameters, PBPK modeling can simulate the dynamic processes of drugs in vivo at the mechanistic level. This enables quantitative prediction of the risk and extent of DDIs. PBPK modeling can be utilized to assess the impact of inhibitors or inducers on drug exposures by alteration of AUC and C_max_, support the development of a rational dose-adjustment strategy, and reduce the number of clinical DDI trials. Especially in complex mechanisms, such as time-dependent inhibition, metabolite involvement, and multi-enzyme co-metabolism, or special populations (e.g., patients with hepatic insufficiency), PBPK modeling can effectively quantify the risk of DDI and promote precise drug use and risk management. Nowadays, it has become an indispensable tool in new drug development and regulatory assessment [34]. In the Gerner et al. article, ruxolitinib (RUX) is metabolized predominantly by CYP3A4 and CYP2C9 and is approved to treat steroid-refractory acute and chronic graft versus host disease (GvHD). The development of the PBPK modeling was utilized for DDI of RUX and posaconazole (POS), a strong CYP3A4 inhibitor. As a result of simulating concomitant administration of POS, the developed PBPK DDI model predicted a 20.5% increase in RUX C_max_ and a 59% increase in AUC_last_, respectively. The calculated DDI ratios for C_max_ and AUC_last_ were 1.21 and 1.59, respectively [35]. Umeharad et al. simulated coadministration with erythromycin and rifampicin, and the results were found to be consistent with clinically observed values within a 20% margin of error [36]. Further simulations showed a 1.94- to 4.31-fold increase in ruxolitinib AUC when combined with fluconazole, a dual inhibitor of CYP3A4 and CYP2C9. Based on these results, the researchers proposed a strategy to reduce the ruxolitinib dose by half in the presence of a strong inhibitor. This PBPK model was used to support the FDA and European Medicines Agency (EMA) updates for ruxolitinib specification, reducing the need for additional clinical DDI studies and demonstrating the value of PBPK modeling in real drug development.

In addition, several studies have systematically demonstrated the value of applying PBPK modeling in PK prediction and DDI risk assessment of natural products. Piperine is a natural alkaloid derived from plants in the piperaceae family, including black pepper and long pepper, and is widely used for food flavoring and traditional herbal medicine compounding. Doses ranging from 5 to 20 mg are commonly used in clinical studies [37]. Literature reports have demonstrated that piperine not only exhibits pharmacological properties such as antioxidant, anti-inflammatory, and digestive stimulation, but has also garnered significant attention as a bio-enhancer capable of augmenting the bioavailability and pharmacokinetic effects of a diverse spectrum of drugs, including rifampicin [38] and docetaxel [39], and dietary phytochemicals, such as resveratrol [17] and curcumin [40, 41].

This enhancement has been attributed to the inhibitory effect of piperine on the activity of the CYP3A4 enzyme, phase II conjugation enzymes (UGT and SULT), and transporters, which would prolong the absorption, half-life of the drug, and enhance the systemic exposure of the drug by decreasing the rate of metabolism in the body [41–43]. To systematically assess the PK properties of piperine and its potential DDI/ food-drug interaction (FDI) risk with CYP3A4 substrates, Lin et al. used the Simcyp PBPK modeling platform to integrate the physicochemical properties of piperine, in vitro metabolism experimental data, and published clinical data from human subjects [44].

After validating the established model using data from healthy Chinese volunteers, further study was conducted to explore the potential DDI risk of piperine when co-administered with 10 CYP3A4 substrate drugs, including carbamazepine, midazolam, clarithromycin, etc. The simulation set up a daily intake of piperine of 20 mg for 7 days, followed by co-administration of the above drugs. Following the DDI guidance that predicted the AUC ratio of a victim drug was ≥ 1.25 in the presence and absence of a studied compound, the results indicated that the AUC ratios of six of these drugs in the piperine-containing to piperine-free conditions exceeded 1.25. Specifically, ritonavir (1.31), nifedipine (1.34), cyclosporine (1.35), triazolam (1.36), alfentanil (1.39), and simvastatin (1.59). In addition, C_max_ of the ten drugs elevated to varying degrees, up to 49%. These results suggested that increased systemic exposure to CYP3A4 substrate drugs may be triggered at normal dietary intake, leading to a potential risk of toxicity or overdose [44].

PBPK modeling also plays a pivotal role in the development of novel natural products, including dietary phytochemicals. For instance, Tam et al. constructed a PBPK/PD model encompassing formononetin, biochanin A, daidzein, and genistein. This model accounted for the variations in drug concentration and the processes of intestinal and hepatic circulation in both humans and animals. Simultaneously, they established a PD kinetic model for bone formation and resorption. Based on the simulation results, a reasonable dosage interval of 5-200 mg per day was proposed, providing a dosage basis for the clinical application of red clover preparations [45].

Furthermore, in the study of curcumin, a PBPK model was developed based on a solid lipid nanoparticle (SLN) formulation. This model was constructed to accurately predict the pharmacokinetic properties of curcumin in healthy volunteers after a single oral dose of 160 mg. The model predicted an overall bioavailability of curcumin of approximately 8%, which was significantly higher than that of crude curcumin powder (< 1%). Moreover, PBPK simulations demonstrated that while the SLN formulation enhanced curcumin absorption, its DDI risk with CYP3A4 substrate drugs such as imatinib and bosutinib remained low. Clinically significant influences were likely only observed at very high doses (> 3.2–6.4 g). These findings further support the application of PBPK modeling in the prediction of natural product-drug interactions [46].

PBPK modeling can also estimate local tissue exposure levels of natural biomolecules. For instance, a PBPK modeling analysis indicated that cichoriin plasma and intracellular concentrations could reach the levels required for anti-SARS-CoV-2 effects following an intravenous dose of 100 mg/kg. Notably, a peak in lung tissues was observed, which supported its feasibility as a potential COVID-19 therapeutic candidate [47].

In contrast to the traditional static DDI model, the PBPK model (dynamic DDI approach) offers a more comprehensive integration of drug absorption, metabolism, and transport processes. It effectively simulates the variations in drug exposures and interactions across diverse populations with varying dosage regimens. The PBPK methodology not only enhances the scientific rigor of early drug screening and clinical trial design but also provides a robust tool to mitigate safety concerns in clinical trials and expedite the development of novel drugs. This approach is particularly well-suited for natural products, characterized by intricate material composition and limited clinical data.

#### Formulation Simulation

In addition to predicting the effects of FIH and DDI, the PBPK modeling can simulate various formulations based on collected data, particularly in simulating different scenarios of oral administration, such as simulating varied drug forms, dosages, and regimens [48]. The development of oral dosage forms remains the preferred strategy for advancing novel chemical entities during drug development. This process can be substantially enhanced by utilizing precise mechanistic models capable of simulating in vivo bioavailability. To effectively optimize oral drug absorption, solubility, dissolution, precipitation, and permeation are the four critical parameters that should be carefully evaluated [10].

In the general workflow of formulation simulation, validated preclinical PBPK models employing in vitro-in vivo correlation (IVIVC) data and dissolution profiles in the GI compartments need to be established prior to simulating human PBPK models. IVIVC is derived from pharmacokinetic data and simulation of in vivo drug dissolution profiles from in vitro dissolution testing, such as Fasted State Simulated Intestinal Fluid (FaSSIF) and Fed State Simulated Intestinal Fluid (FeSSIF). These correlations facilitate the prediction of in vivo concentration-time profiles based on in vitro dissolution behavior of various formulations. This approach provides a valuable tool for optimizing oral dosage forms while reducing the reliance on extensive in vivo animal studies or clinical trials, further eliminating superfluous cost and time [49, 50].

Investigation of drug release profiles is a crucial process in drug formulation [51]. Modified release (MR) formulations, such as delayed, controlled, and targeted release, modify drug release timing and site compared to conventional immediate release forms. The zero-order release, which maintains constant drug levels in blood or tissues, is primarily achieved through osmotic systems; however, most other formulations (e.g., matrix, reservoir) follow first-order kinetics, which complicates in vivo prediction. Additional challenges arise from formulation factors (e.g., polymers, manufacturing variability) and physiological variability (e.g., gastrointestinal transit, pH, food effects). However, the employment of modeling tools and IVIVC approaches assists in overcoming these challenges. Modeling tools like Phoenix WinNonlin facilitate deconvolution-based target dissolution profiling, while PBPK platforms such as GastroPlus and Simcyp incorporate physiological factors for predicting systemic exposure and PK/PD relationships [52]. Furthermore, GastroPlus incorporates the advanced compartmental absorption and transit (ACAT) model, which deconvolutes the in vivo intestinal release profile [53]. Next, we present two studies using the PBPK software for formulation simulation.

Lukacova et al. investigated the value of mechanistic simulations in predicting the in vivo behavior of modified release (MR) formulations, particularly in the context of PK and PD. The study utilizes the GastroPlus software suite to develop and calibrate comprehensive models for adinazolam and metoprolol based on in vitro, immediate release (IR) oral, and intravenous data. The adinazolam model successfully predicts PD profiles for an existing MR formulation and demonstrates the potential for designing new formulations with desired onset and duration of action. The metoprolol model provides valuable insights into the factors influencing the exposure of MR formulations with different release rates, highlighting the complexities of gastrointestinal absorption, particularly in the colon, and the potential limitations of relying solely on in vitro dissolution data. The study emphasizes the advantages of mechanistic modeling over traditional IVIVC by explicitly describing all processes affecting drug bioavailability and linking them to physiological parameters [54].

In Otsuka et al. study, the authors successfully predicted the in vivo performance of furosemide, a challenging Biopharmaceutical Classification System (BCS) class IV drug, from both IR and MR formulations using an in vitro–in–silico–in–vivo (IVISIV) approach. The PBPK model, coupled with biorelevant dissolution data (FaSSIF and FeSSIF), was instrumental in identifying the key factors influencing drug absorption for each formulation type. Gastric emptying and absorption rate are critical for IR tablets, while release rate in the small intestine also becomes a significant factor for MR capsules. This approach has the potential to facilitate formulation development by identifying critical parameters and helping to understand the sources of variability in drug absorption [55].

PBPK modeling has become a powerful tool in both innovative and generic drug development, aiding in formulation design, regulatory submissions, and clinical decision-making. Its applications span MR formulations, biowaivers (even for BCS II drugs), particle size distribution (PSD) specification, and prediction of drug performance across various administration routes, including ocular, pulmonary, and transdermal. These models are also increasingly used to simulate pharmacokinetics in special populations where clinical trials may be challenging. Future advancements will rely on improving model assumptions, incorporating high-quality measured input data, and addressing transporter kinetics. Ultimately, PBPK models will help reduce reliance on in vivo studies and accelerate access to safe, effective, and affordable medicines.

#### Extension To Pediatric and Special Populations

For special populations, the PBPK modeling is an attractive tool for determining drug regimens in pediatric, geriatric, or organ-impairment patients in late-stage drug development, particularly in Phase 2 and Phase 3 clinical trials [56]. PBPK modeling holds a significant advantage in regulatory applications during drug development. Its primary function is to enhance the evidence base for special population drug dosing recommendations by effectively utilizing existing animal or human PK data.

Previous studies have demonstrated that the PBPK model can be instrumental in optimizing the utilization of various drugs in pediatric patients [57]. Adhering to the pediatric regulatory framework of the European Union (EU), pediatric clinical trials can span several years, potentially exceeding that duration. Furthermore, the costs associated with a single drug inclusion in a formal pediatric treatment plan are substantial [58]. Typically, the drug-specific data and physiological data are segregated in the PBPK model. This enables the PK simulation of a drug in any virtual population, such as a pediatric model, by utilizing a compound model [59]. This approach presents a viable pathway for supporting dosage selection in children’s clinical care. Consequently, the PBPK model emerges as an efficient and cost-effective method for simulating PK data and providing dosing regimens tailored to various age groups. Subsequently, we will provide four studies to demonstrate the application of PBPK modeling on pediatric and special populations.

Maglalang et al.’s study used the PBPK model as a framework and simulated the PK data from two prospective pediatric trials. The age range was from 1 month to 19 years, and some of the subjects were obese. This study evaluated the influence of renal maturation level on levetiracetam disposition using the PK data from clinical studies within the PBPK model. Subjects in their study were neonates and children younger than 2 years old. The analysis of AUC and C_min_ median values was achieved by the PBPK model, and the difference between the FDA pediatric review of levetiracetam and the median values they obtained was acceptable, under similar doses. The levetiracetam disposition in pediatric populations was characterized by PBPK model fitting, which filled the gap in identifying the drug dosage in children with obesity, neonates, and younger children with seizures [60].

In Guimarães et al.’s study, they aimed to build a PBPK model developed in Simcyp, coupled with the ADAM model for oral absorption and age-appropriate in vitro dissolution data, to evaluate montelukast pediatric formulations through two case studies. The goal is to support decision-making and biopharmaceutics understanding during pediatric drug development. Study 1 focused on extrapolating the performance of montelukast chewable tablets from adults to children aged 2 to 5 years. The PBPK model successfully described adult exposure in the fasted state, meeting validation criteria with the absolute average fold error (AAFE) ≤ 1.52. When applied to children (2–5 years), simulations also met validation criteria, with AAFE values ranging from 1.52 to 1.75. The study found that using a two-stage fasted gastric to fasted intestinal dissolution profile as input in the model resulted in the most successful prediction in both populations. Regarding bioavailability, the model estimated the fraction absorbed from chewable tablets to be approximately 80% in adults, consistent with reported values. In children, the simulated bioavailability was slightly lower, approximately 68%. Parameter sensitivity analysis indicated that in vivo dissolution in children is likely fast but incomplete, needing around 60% dissolution for optimal predictions, contrasting with the 80–100% needed for adults. Case Study 2 investigated the impact of co-administering montelukast granules with vehicles (formula or applesauce) on exposure in infants aged 1 to 24 months. Fasted state simulations were found to be not predictive, particularly for older infants, and did not meet the validation criteria (AAFE ≤ 2). In contrast, coupling dissolution profiles simulating co-administration under fed intestinal conditions (FaG/FeI) with a fed state PBPK model successfully described infant drug performance, meeting validation criteria (AAFE ≤ 2) across subgroups: 1–3 months with formula (AAFE 1.37), 3–6 months with applesauce (AAFE 1.70), and 6–24 months with applesauce (AAFE 1.49). Simulated bioavailability in infants was estimated at approximately 76% for the 1–3 months group (formula) and approximately 58% for the 3–24 months group (applesauce). Sensitivity analysis suggested likely fast but incomplete in vivo dissolution in infants, with best C_max_ predictions achieved with dissolution extent between 60 and 80% for younger infants and 40–60% for older infants [61].

PBPK modeling can analyze additional effects such as co-medication, ethnicity, renal impairment, or obesity on the drug’s PK, which are commonly observed in geriatric populations [62, 63]. Elderly individuals typically exhibit distinct PK characteristics compared to younger individuals due to organ and bodily function deterioration. For instance, the oral clearance of theophylline in elderly individuals aged between 62 and 93 was approximately 54% lower compared to those aged between 21 and 24. In this study, internal validation was conducted using height, weight, cardiac output, and serum creatinine values. To assess the accuracy of physiological characteristics, 5% and 95% percentiles of these variables were compared with observed data. The results demonstrated that the discrepancies exceeding 95% predicted AUC, C_max_, and CL values were within an acceptable range [64].

Furthermore, geriatric individuals with organ degeneration and dysfunction constitute one of the most vulnerable populations susceptible to COVID-19. Wang et al.’s study focuses on the evaluation of dosing recommendations for baricitinib and tofacitinib as treatment options. The study subjects included geriatric patients with chronic renal impairment. The PBPK model was established for age-dependent simulations, utilizing the plasma concentration-time profile and pertinent PK parameters to assess the efficacy of the drugs. Based on the data obtained, the majority of the AUC ratios were less than 1.84, thereby confirming that the current dosage regimen based on glomerular filtration rate (GFR) and in the presence of a potent hOAT3 inhibitor is appropriate for geriatric patients [65].

PBPK modeling is a powerful tool for determining safe and effective drug dosages for specialized populations, such as children and the elderly, particularly during the late stages of drug development. This method is both efficient and cost-effective, enabling researchers to simulate drug behavior in virtual patient groups by integrating drug and physiological data. Consequently, it supports dosage decisions and diminishes the necessity for extensive clinical trials. Studies, including those on levetiracetam and montelukast in children and baricitinib in geriatric patients, demonstrate how PBPK models can predict drug behavior and facilitate optimal dosing in these vulnerable populations. Notably, these models account for factors such as age, obesity, and organ impairment. Ultimately, PBPK modeling provides a quantitative method for dosage adjustment and assessing drug risks for diverse patient cohorts [66].

### PBPK Modeling of Dietary Phytochemicals

Advancements in natural medicine research have enhanced the attention given to the PK characteristics of phytochemicals, the bioactive components found in medicinal plants. PK/PD studies of phytochemicals have elucidated the correlation between their in vivo behavior and the PD effects, thereby providing empirical evidence that supports the development and clinical translation of phytopharmaceuticals. In our published articles, we demonstrated the PK/PD profiles of well-known phytochemicals, such as sulforaphane [67], curcumin [68–70], ursolic acid [71], and 3,3’-diindolylmethane [72] in the rat model and in human subjects. Furthermore, we explored the PBPK models of curcumin to provide insights into clinical applications [68, 70].

Generally, due to the intricate nature of natural products’ components, the diversity of metabolic pathways, and the presence of species and population variations, traditional non-compartmental or compartmental PK modeling approaches predominantly rely on empirical mathematical equations to elucidate the concentration-effect correlation of drugs in vivo; however, their simplified structure frequently lacks consideration of the actual physiological structure of tissues and organs, the distribution of metabolic enzymes, and the variations within specific populations. Consequently, the applicability of these models to numerous dietary phytochemical compounds with intricate compositions may be limited [73].

In contrast, the PBPK model, grounded in systematic anatomy and physiological parameters, can simulate the ADME properties of drugs across diverse tissues and organs, making it particularly suited for intricate and variable phytochemicals [74]. As a systematic modeling approach that integrates the physicochemical properties of drugs, physiological parameters, enzymology, and transport mechanisms, the PBPK modeling has garnered widespread application in predicting in vivo behavior, optimizing dosages, extrapolating to specific populations, and conducting studies on drug-natural product interactions [10]. Several notable studies illustrating the multidimensional application of the PBPK model in natural product research are presented in the following context, spanning from single-component to multi-component, from healthy populations to pathological states, and from fundamental metabolic mechanisms to clinical extrapolation.

Mitragynine (the primary psychoactive alkaloid of kalaurenine), the PBPK model successfully predicted its blood concentration-time profile in rats and humans by incorporating the mechanisms of brain P-glycoprotein (Pgp) transport, CYP3A4 metabolism, and adipose tissue distribution restriction. These mechanisms provided the foundation for the subsequent optimization of the topical delivery of analgesic and antidepressant medications [75]. Another study utilizing deoxypodophyllotoxin (DPT), a cyclolignan compound, integrated PBPK and PD models to predict its plasma, tumor, and normal tissue exposure in tumor-bearing mice. This approach accurately simulated tumor growth inhibition curves, offering a novel strategy to bridge the gap between in vitro cytotoxicity data and in vivo efficacy. Consequently, it expedites the screening of anticancer drugs and the design of dosage forms for antitumor natural substance development [76].

Li et al. developed a systematic pharmacokinetic model (PBPK) for schaftoside, a prominent flavonoid glycoside found in the total flavonoids of *Desmodium styracifolium* (TFDS). This model enabled effective cross-species extrapolation between animal and human pharmacology [77]. The model was initially developed by integrating PK data from intravenous and oral administration of TFDS in rats. It was discovered that schaftoside exhibited poor metabolism in rat and human liver microsomes, primarily excreted in the form of a prototypic drug via urine (~ 55%) and bile (~ 25%). Based on these metabolic and excretory characteristics, the model was established as a low metabolic clearance type, and complex enzymatic kinetics were not introduced. The established rat model successfully predicted the plasma concentration-time curve of schaftoside, with C_max_ and AUC₀₋ₜ predicted values less than 1.5-fold in the fold error (FE) compared to measured values, fulfilling the validation criteria of the PBPK model. Subsequently, the model was extrapolated to a healthy population using PK-Sim and validated with multi-dose clinical data from four subjects, exhibiting an excellent model fit. Further extension of this adult PBPK model to an elderly population (71–80 years old) and patients with varying stages of chronic kidney disease (CKD) revealed no significant difference in drug exposure between the elderly and healthy adults. Conversely, patients with CKD stages 3, 4, and 5 exhibited a 3.41-fold, 12.32-fold, and 23.77-fold increase in C_max_, respectively. This suggests that clearance is dependent on renal function, indicating that the model may provide a quantitative basis for dose adjustment in this patient population [77].

In addition to single herbal active ingredients, the PBPK model can be further extended to phytochemical mixture systems, such as the complex tea catechin mixture (TCM). A notable example of this is the PBPK modeling study conducted on green tea [78]. The model was developed from the rat PBPK model, and epigallocatechin gallate (EGCG) was selected as a representative to confirm the consistency in vivo behavior of EGCG between the two dosage forms by comparing the pharmacokinetic data after the ingestion of pure EGCG and the EGCG fraction in Polyphenon E (PE) in rats. The model validation data showed mean absolute prediction errors (MAPEs) of 17.4% and 14.6%, demonstrating good predictive accuracy. Subsequently, the model was extrapolated to humans and successfully reproduced the blood concentration profiles observed in several clinical studies by substituting physiological parameters, distribution coefficients, absorption, and transport parameters with human values. The PBPK model of EGCG was further integrated with epicatechin gallate (ECG) and epicatechin (EC) models to construct a comprehensive TCM hybrid model. Validated by multiple clinical datasets, the hybrid PBPK model can simultaneously and accurately describe the blood drug dynamics of the three tea catechins after ingestion of green tea extracts in humans with MAPEs ranging from 20 to 30%. The model output exhibits a linear relationship with the dose, indicating excellent dose prediction capabilities [79–81]. This study effectively demonstrated that the PBPK model can be employed for comprehensive modeling of components within natural mixtures, overcoming the conventional limitation of modeling solely based on a single component.

Additionally, PBPK studies of resveratrol demonstrate a pioneering approach to applying PBPK models to simulate intricate metabolic processes, such as phase II metabolism and the enterohepatic cycle. Han et al. synthesized a comprehensive PBPK model encompassing twelve organ compartments. This model integrates multidimensional data on in vitro protein binding rate, intestinal/hepatic S9 fraction metabolism, in situ intestinal perfusion, and in vivo PK and excretion. Notably, the model simulates glucuronidation/sulfation processes and the enterohepatic cycle, enabling a more accurate creation of a systemic PBPK model. The initial modeling was conducted using animal data. Through a systematic optimization of parameters such as absorbed fraction (F_abs_), first-order absorption rate constant (k_a_), maximal metabolic rate (V_max_), Michaelis–Menten constant (K_m_), and other parameters, the model achieved an accuracy of AUC_last_ ratios ranging from 0.761 to 0.999 and C_max_ ratios ranging from 0.676 to 0.922 by comparing predicted and observed data. Furthermore, the model accurately predicted the blood concentration dynamics in both single-dose and multi-dose regimens. In the single-dose scenario, the model predicted the dynamics for both 200 mg and 400 mg oral doses in humans. Similarly, in the multi-dose regimen, the model predicted the dynamics for a thrice-daily oral dose of 200 mg for four days and a twice-daily oral dose of 2000 mg for seven days. Notably, the simulation results demonstrated that the enterohepatic circulation of resveratrol significantly contributes to drug exposure in rats, while its role in humans is less pronounced. This observation underscores the species-specific differences in metabolic pathways [82].

PBPK modeling research comprehensively encompasses a diverse spectrum of phytochemicals, ranging from individual components to mixed formulations. Furthermore, it effectively predicts pharmacokinetics across various populations, including healthy individuals, the elderly, and patients with renal insufficiency. Through meticulous system construction, parameter optimization, model validation, and population extrapolation, PBPK modeling effectively addresses the complexities inherent in the composition and metabolism of natural products. This approach provides robust theoretical support and practical foundation for individualized medication and natural health product development, surpassing conventional PK methodologies. Notably, this research direction suggests that PBPK modeling will assume a more extensive and profound role in the future, particularly in the domains of plant-derived drug dosage optimization, DDI risk prediction, and the construction of efficacy-exposure relationships.

Although PBPK modeling offers numerous advantages over conventional PK modeling in the application of phytochemicals, there are certain limitations that warrant consideration. Firstly, the identification of the primary and representative phytochemicals. Secondly, the incorporation of internal interactions, such as metabolism and transporter kinetics. Lastly, the availability of limited preclinical and clinical data in comparison to synthetic drugs. For instance, PBPK modeling necessitates the incorporation of a multitude of organism parameters, drug parameters, and in vitro and in vivo parameters pertaining to drug metabolism. These parameters include the volume and blood flow rates of various tissues and organs, drug solubility, pK_a_, plasma-protein binding rates, and enzyme and transporter characteristics. Consequently, PBPK modeling is more appropriate for investigating individual compounds with well-defined structures and principal components with substantial concentrations in natural drugs to further elucidate the individual and synergistic effects of multiple components in pharmacokinetic research [73].

## Conclusion

PBPK modeling is a powerful, advanced, and mechanistic computational tool that is gaining significant acceptance from regulatory bodies, including the FDA. In contrast to traditional PK methods that may lack physiological detail, PBPK modeling integrates drug properties with organism-specific physiological parameters (such as organ volumes, blood flow) to predict drug behavior in major body compartments, offering high physiological realism.

PBPK modeling is widely applied throughout drug development. It aids lead optimization and candidate evaluation in the discovery phase by predicting human PK from preclinical data and identifying ADME issues. A key application is the prediction of drug-drug interactions by simulating complex in vivo processes, supporting dose adjustments, and potentially reducing the need for clinical DDI trials. PBPK is also valuable for formulation simulation, particularly for oral and modified release forms, helping optimize bioavailability and predict performance based on in vitro data, thus decreasing the need for extensive in vivo studies. Furthermore, it is a crucial tool for extending drug knowledge to pediatric and special populations (elderly, organ impairment, obesity) by simulating drug behavior in virtual groups, supporting safe and effective dosage determination efficiently and cost-effectively, thereby reducing the necessity for clinical trials in these vulnerable populations.

Specifically with dietary phytochemicals and natural products, PBPK modeling is well-suited for their complexity and variability. Integration of physiological and structural parameters, PBPK models can simulate ADME properties across tissues and organs, providing theoretical support for dosage optimization, DDI risk prediction, and individualized natural product development. Examples include studies on schaftoside, tea catechins, and resveratrol, demonstrating its utility for cross-species extrapolation, dose adjustment in diseased populations, and modeling intricate metabolic processes. However, challenges remain, such as identifying representative bioactive components, modeling complex interactions, and limited data availability compared to synthetic drugs.

In summary, PBPK modeling is presented as a dynamic and quantitative methodology that effectively integrates pharmacokinetic properties across diverse populations and therapeutic treatments. Its application across various stages of drug development and its adaptability to intricate substances, including natural products, underscore its growing significance in bolstering scientific rigor, accelerating drug discovery, and guaranteeing patient safety.

## Data Availability

No datasets were generated or analysed during the current study.

## Abbreviations

AAFE: Absolute average fold error
ADME: Absorption, distribution, metabolism and excretion
AUC: Area under the plasma concentration-time curve
AUClast: AUC from time zero to time of last measurable concentration
BCS: Biopharmaceutical classification system
CKD: Chronic kidney disease
CL: Apparent total body clearance of the drug from plasma
Cmax: Maximum plasma drug concentration
Cmin: Minum plasma drug concentration
DDI: Drug-drug interactions
DPT: Deoxypodophyllotoxin
EC: Epicatechin
ECG: Epicatechin gallate
EGCG: Epigallocatechin gallate
EMA: European medicines agency
Fabs: Absorbed fraction
FaSSIF: Fasted state simulated intestinal fluid
FDA: Food and drug administration
FDI: Food-drug interactions
FE: Fold error
FeSSIF: Fed state simulated intestinal fluid
FIH: First-in-human
fu: Fraction of the drug unbound
GFR: Glomerular filtration rate
GI: Gastrointestinal
GvHD: Graft versus host disease
hOAT3: Human organic anion transporter 3
IDR: Indirect response
IR: Immediate release
IVISIV: In vitro–in–silico–in–vivo
IVIVC: In vitro-in vivo correlation
IVIVE: In vitro-in vivo extrapolation
Ka: First-order absorption rate constant
Km: Michaelis–menten constant
Kp: Tissue-plasma partition coefficient
LogD: Logarithm of the distribution coefficient
LogP: Logarithm of the partition coefficient
LPS: Lipopolysaccharide
MAPEs: Mean absolute prediction errors
MR: Modified release
MW: Molecular weight
PBPK: Physiologically-based pharmacokinetic
Pgp: P-glycoprotein
PK: Pharmacokinetic
PK/PD: Pharmacokinetic/ pharmacodynamic
pKa: Acid dissociation constants
POS: Posaconazole
PSD: Particle size distribution
QSP: Quantitative systems pharmacology
RUX: Ruxolitinib
SLN: Solid lipid nanoparticle
SULT: Sulfotransferase
T1/2: Elimination half-life
TCM: Tea catechin mixture
TFDS: Total flavonoids of Desmodium styracifolium
Tmax: Time to reach cmax following drug administration
UGT: UDP-glucuronosyltransferase
Vd: Apparent volume of distribution
Vmax: Maximal metabolic rate

## References

[1] Jusko WJ. Moving from basic toward systems pharmacodynamic models. J Pharm Sci. 2013;102(9):2930–40. 23681608 10.1002/jps.23590 PMC3743951

[2] Clarelli F, et al. Multi-scale modeling of drug binding kinetics to predict drug efficacy. Cell Mol Life Sci. 2020;77(3):381–94. 31768605 10.1007/s00018-019-03376-y PMC7010620

[3] Asin-Prieto E, Rodriguez-Gascon A, Isla A. Applications of the pharmacokinetic/pharmacodynamic (PK/PD) analysis of antimicrobial agents. J Infect Chemother. 2015;21(5):319–29. 25737147 10.1016/j.jiac.2015.02.001

[4] Rowland M, Peck C, Tucker G. Physiologically-based pharmacokinetics in drug development and regulatory science. Annu Rev Pharmacol Toxicol. 2011;51:45–73. 20854171 10.1146/annurev-pharmtox-010510-100540

[5] Ezuruike U, et al. Guide to development of compound files for PBPK modeling in the simcyp population-based simulator. CPT: Pharmacometrics Syst Pharmacol. 2022;11(7):805–21. 35344639 10.1002/psp4.12791 PMC9286711

[6] Cao Y, Jusko WJ. Applications of minimal physiologically-based Pharmacokinetic models. J Pharmacokinet Pharmacodyn. 2012;39(6):711–23. 23179857 10.1007/s10928-012-9280-2 PMC3539784

[7] Tsamandouras N, Rostami-Hodjegan A, Aarons L. Combining the ‘bottom up’ and ‘top down’ approaches in Pharmacokinetic modelling: fitting PBPK models to observed clinical data. Br J Clin Pharmacol. 2015;79(1):48–55. 24033787 10.1111/bcp.12234 PMC4294076

[8] Lin W, et al. Applications, challenges, and outlook for PBPK modeling and simulation: A regulatory, industrial and academic perspective. Pharm Res. 2022;39(8):1701–31. 35552967 10.1007/s11095-022-03274-2

[9] Jones HM, et al. A novel strategy for physiologically based predictions of human pharmacokinetics. Clin Pharmacokinet. 2006;45(5):511–42. 16640456 10.2165/00003088-200645050-00006

[10] Kuepfer L, et al. Applied concepts in PBPK modeling: how to build a PBPK/PD model. CPT Pharmacometrics Syst Pharmacol. 2016;5(10):516–31. 27653238 10.1002/psp4.12134 PMC5080648

[11] Bhattacharya T et al. Role of phytonutrients in nutrigenetics and nutrigenomics perspective in curing breast cancer. Biomolecules, 2021;11(8). 10.3390/biom11081176 PMC8394348 34439842

[12] Liu RH. Health-promoting components of fruits and vegetables in the diet. Adv Nutr. 2013;4(3):S384–92. 10.3945/an.112.003517 PMC3650511 23674808

[13] Chou PJ, et al. Epigenetics of dietary phytochemicals in cancer prevention: fact or fiction. Cancer J. 2024;30(5):320–8. 39312452 10.1097/PPO.0000000000000742 PMC11573353

[14] Wu R, et al. Redox signaling, mitochondrial metabolism, epigenetics and redox active phytochemicals. Free Radic Biol Med. 2022;179:328–36. 33359432 10.1016/j.freeradbiomed.2020.12.007 PMC8222414

[15] Kotecha R, Takami A, Espinoza JL. Dietary phytochemicals and cancer chemoprevention: a review of the clinical evidence. Oncotarget. 2016;7(32):52517–29. 27232756 10.18632/oncotarget.9593 PMC5239570

[16] Shannar A, et al. Pharmacodynamics (PD), pharmacokinetics (PK) and PK-PD modeling of NRF2 activating dietary phytochemicals in cancer prevention and in health. Curr Pharmacol Rep. 2025;11(1):6. 39649473 10.1007/s40495-024-00388-6 PMC11618211

[17] Johnson JJ, et al. Enhancing the bioavailability of Resveratrol by combining it with Piperine. Mol Nutr Food Res. 2011;55(8):1169–76. 21714124 10.1002/mnfr.201100117 PMC3295233

[18] Sanna V, et al. Resveratrol-loaded nanoparticles based on poly(epsilon-caprolactone) and poly(D,L-lactic-co-glycolic acid)-poly(ethylene glycol) blend for prostate cancer treatment. Mol Pharm. 2013;10(10):3871–81. 23968375 10.1021/mp400342f PMC4100701

[19] Balata GF, et al. Self-emulsifying drug delivery systems as a tool to improve solubility and bioavailability of Resveratrol. Drug Des Devel Ther. 2016;10:117–28. 26792979 10.2147/DDDT.S95905 PMC4708959

[20] Utembe W, et al. Current approaches and techniques in physiologically based Pharmacokinetic (PBPK) modelling of nanomaterials. Nanomaterials. 2020;10(7):1267. 32610468 10.3390/nano10071267 PMC7407857

[21] Chetty M, et al. Physiologically based Pharmacokinetic modelling to guide drug delivery in older people. Adv Drug Deliv Rev. 2018;135:85–96. 30189273 10.1016/j.addr.2018.08.013

[22] Li M, et al. Physiologically based Pharmacokinetic modeling of PLGA nanoparticles with varied mPEG content. Int J Nanomed. 2012;7:1345–56. 10.2147/IJN.S23758 PMC3299578 22419876

[23] Khalil F, Läer S. Physiologically based Pharmacokinetic modeling: methodology, applications, and limitations with a focus on its role in pediatric drug development. Volume 2011. BioMed Research International; 2011;907461:1. 10.1155/2011/907461 PMC3118302 21716673

[24] Jones H, Rowland-Yeo K. Basic concepts in physiologically based Pharmacokinetic modeling in drug discovery and development. CPT Pharmacometrics Syst Pharmacol. 2013;2(8):e63. 23945604 10.1038/psp.2013.41 PMC3828005

[25] Lin Z, et al. A computational framework for interspecies pharmacokinetics, exposure and toxicity assessment of gold nanoparticles. Nanomed (Lond). 2016;11(2):107–19. 10.2217/nnm.15.177 26653715

[26] Zazo H et al. Physiologically based Pharmacokinetic (PBPK) model of gold Nanoparticle-Based drug delivery system for stavudine biodistribution. Pharmaceutics, 2022;14(2). 10.3390/pharmaceutics14020406 PMC8875329 35214138

[27] Santos LGA, et al. Real-world application of physiologically based Pharmacokinetic models in drug discovery. Drug Metab Dispos. 2025;53(1):100015. 39884820 10.1124/dmd.122.001036

[28] Zhuang X, Lu C. PBPK modeling and simulation in drug research and development. Acta Pharm Sin B. 2016;6(5):430–40. 27909650 10.1016/j.apsb.2016.04.004 PMC5125732

[29] Barcelos MP et al. Lead Optimization in Drug Discovery, in Research Topics in Bioactivity, Environment and Energy: Experimental and Theoretical Tools, C.A. Taft and S.R. de Lazaro, Editors. Springer International Publishing: Cham. 2022;481–500.

[30] Hughes J, et al. Principles of early drug discovery. Br J Pharmacol. 2011;162(6):1239–49. 21091654 10.1111/j.1476-5381.2010.01127.x PMC3058157

[31] Huang J, et al. Systematic prediction of pharmacodynamic drug-drug interactions through protein-protein-interaction network. PLoS Comput Biol. 2013;9(3):e1002998. 23555229 10.1371/journal.pcbi.1002998 PMC3605053

[32] Han K, et al. A review of approaches for predicting drug–drug interactions based on machine learning. Front Pharmacol. 2022;12:814858. 35153767 10.3389/fphar.2021.814858 PMC8835726

[33] Tornio A, et al. Clinical studies on drug–drug interactions involving metabolism and transport: methodology, pitfalls, and interpretation. Volume 105. Clinical Pharmacology & Therapeutics 2019;(6):1345–61. 10.1002/cpt.1435 PMC6563007 30916389

[34] Zhuang X, Lu C. PBPK modeling and simulation in drug research and development. Acta Pharm Sinica B. 2016;6(5):430–40. 10.1016/j.apsb.2016.04.004 PMC5125732 27909650

[35] Gerner B, et al. A physiologically-based Pharmacokinetic model of ruxolitinib and posaconazole to predict CYP3A4-mediated drug–drug interaction frequently observed in graft versus host disease patients. Pharmaceutics. 2022;14(12):2556. 36559050 10.3390/pharmaceutics14122556 PMC9785192

[36] Umehara K, et al. Drug-drug interaction (DDI) assessments of ruxolitinib, a dual substrate of CYP3A4 and CYP2C9, using a verified physiologically based Pharmacokinetic (PBPK) model to support regulatory submissions. Drug Metabolism Personalized Therapy. 2019;34(2):20180042. 10.1515/dmpt-2018-0042 31145690

[37] Singletary K. Black pepper: overview of health benefits. Nutr Today. 2010;45(1):43–7.

[38] Balakrishnan V, Varma S, Chatterji D. Piperine augments transcription inhibitory activity of rifampicin by severalfold in Mycobacterium smegmatis. Curr Sci. 2001;80(10):1302–5.

[39] Makhov P, et al. Co-administration of Piperine and docetaxel results in improved anti-tumor efficacy via Inhibition of CYP3A4 activity. Prostate. 2012;72(6):661–7. 21796656 10.1002/pros.21469 PMC3208085

[40] Rinwa P, Kumar A. Piperine potentiates the protective effects of Curcumin against chronic unpredictable stress-induced cognitive impairment and oxidative damage in mice. Brain Res. 2012;1488:38–50. 23099054 10.1016/j.brainres.2012.10.002

[41] Gorgani L, et al. Piperine—the bioactive compound of black pepper: from isolation to medicinal formulations. Compr Rev Food Sci Food Saf. 2017;16(1):124–40. 33371546 10.1111/1541-4337.12246

[42] Lee SH, et al. Piperine-mediated drug interactions and formulation strategy for piperine: recent advances and future perspectives. Expert Opinion on Drug Metabolism & Toxicology; 2018;14(1):43–57. 10.1080/17425255.2018.1418854 29250980

[43] Tiwari A, Mahadik KR, Gabhe SY. Piperine: A comprehensive review of methods of isolation, purification, and biological properties. Med Drug Discovery. 2020;7:100027.

[44] Lin F, et al. Predicting Food–Drug interactions between Piperine and CYP3A4 substrate drugs using PBPK modeling. Int J Mol Sci. 2024;25(20):10955. 39456737 10.3390/ijms252010955 PMC11506926

[45] Tam YK, et al. Development of a phytoestrogen product for the prevention or treatment of osteoporosis using red clover. Google Patents 2012.

[46] Adiwidjaja J, Boddy AV, McLachlan AJ. Physiologically-based Pharmacokinetic predictions of the effect of Curcumin on metabolism of Imatinib and bosutinib: in vitro and in vivo disconnect. Pharm Res. 2020;37:1–16. 10.1007/s11095-020-02834-8 32529309

[47] Rivero-Segura NA, Gomez-Verjan JC. In Silico screening of natural products isolated from Mexican herbal medicines against COVID-19. Biomolecules. 2021;11(2):216. 33557097 10.3390/biom11020216 PMC7913859

[48] Ahmad A, et al. IMI - Oral biopharmaceutics tools project - Evaluation of bottom-up PBPK prediction success part 4: prediction accuracy and software comparisons with improved data and modelling strategies. Eur J Pharm Biopharm. 2020;156:50–63. 32805361 10.1016/j.ejpb.2020.08.006

[49] Dressman JB, et al. Dissolution testing as a prognostic tool for oral drug absorption: immediate release dosage forms. Pharm Res. 1998;15:11–22. 9487541 10.1023/a:1011984216775

[50] Cardot J, Beyssac E, Alric M. vitro-in vivo correlation: importance of dissolution in IVIVC. Dissolution Technol. 2007;14(1):15.

[51] Hassan S, Zhang YS. Chap. 10 - Microfluidic technologies for local drug delivery, in Microfluidics for Pharmaceutical Applications, H.A. Santos, D. Liu, and H. Zhang, Editors. William Andrew Publishing. 2019;281–305.

[52] Kaur G, et al. Oral controlled and sustained drug delivery systems: concepts, advances, preclinical, and clinical status, in Drug targeting and stimuli sensitive drug delivery systems. Elsevier; 2018;567–626.

[53] Lukacova V, Woltosz WS, Bolger MB. Prediction of modified release pharmacokinetics and pharmacodynamics from in vitro, immediate release, and intravenous data. Aaps J. 2009;11(2):323–34. 19430911 10.1208/s12248-009-9107-2 PMC2691467

[54] Lukacova V, Woltosz WS, Bolger MB. Prediction of modified release pharmacokinetics and pharmacodynamics from in vitro, immediate release, and intravenous data. AAPS J. 2009;11:323–34. 19430911 10.1208/s12248-009-9107-2 PMC2691467

[55] Otsuka K, et al. Prediction of in-vivo Pharmacokinetic profile for immediate and modified release oral dosage forms of Furosemide using an in-vitro–in-silico–in-vivo approach. J Pharm Pharmacol. 2015;67(5):651–65. 25644429 10.1111/jphp.12365

[56] Suri A, et al. Physiologically based and population PK modeling in optimizing drug development: A predict-learn-confirm analysis. Clin Pharmacol Ther. 2015;98(3):336–44. 26031410 10.1002/cpt.155 PMC5039936

[57] Freriksen JJM, et al. Physiologically based Pharmacokinetic (PBPK) Model-Informed dosing guidelines for pediatric clinical care: A pragmatic approach for a special population. Paediatr Drugs. 2023;25(1):5–11. 36201128 10.1007/s40272-022-00535-w PMC9534738

[58] Commission E. State of paediatric medicines in the EU—10 years of the EU paediatric regulation. 2017:2022.

[59] Yellepeddi V, et al. State-of-the-Art review on physiologically based Pharmacokinetic modeling in pediatric drug development. Clin Pharmacokinet. 2019;58(1):1–13. 29777528 10.1007/s40262-018-0677-y

[60] Maglalang PD, et al. Application of physiologically based Pharmacokinetic modeling to characterize the effects of age and obesity on the disposition of Levetiracetam in the pediatric population. Clin Pharmacokinet. 2024;63(6):885–99. 38814425 10.1007/s40262-024-01367-2 PMC11225543

[61] Guimarães M, Vertzoni M, Fotaki N. Performance evaluation of Montelukast pediatric formulations: part II - a PBPK modelling approach. Aaps J. 2022;24(1):27. 35013803 10.1208/s12248-021-00662-1 PMC8816611

[62] Xu J, et al. Physiologically based Pharmacokinetic modeling and dose adjustment of Teicoplanin in pediatric patients with renal impairment. J Clin Pharmacol. 2022;62(5):620–30. 34761398 10.1002/jcph.2000

[63] Ford JL, et al. Physiologically based Pharmacokinetic modeling of Metformin in children and adolescents with obesity. J Clin Pharmacol. 2022;62(8):960–9. 35119103 10.1002/jcph.2034 PMC9288496

[64] Cui C, et al. Development of a physiologically based Pharmacokinetic (PBPK) population model for Chinese elderly subjects. Br J Clin Pharmacol. 2021;87(7):2711–22. 33068053 10.1111/bcp.14609 PMC8359847

[65] Wang Z, Chan ECY. Physiologically-Based Pharmacokinetic modelling to investigate baricitinib and Tofacitinib dosing recommendations for COVID-19 in geriatrics. Clin Pharmacol Ther. 2022;112(2):291–6. 35380176 10.1002/cpt.2600 PMC9087009

[66] Zhang M, et al. Prediction of Pyrotinib exposure based on physiologically-based Pharmacokinetic model and endogenous biomarker. Front Pharmacol. 2022;13:972411. 36210839 10.3389/fphar.2022.972411 PMC9543720

[67] Wang H, et al. Pharmacokinetics and pharmacodynamics of phase II drug metabolizing/antioxidant enzymes gene response by anticancer agent Sulforaphane in rat lymphocytes. Mol Pharm. 2012;9(10):2819–27. 22931102 10.1021/mp300130k PMC3580178

[68] Wang L, et al. Pharmacokinetics and pharmacodynamics of three oral formulations of Curcumin in rats. J Pharmacokinet Pharmacodyn. 2020;47(2):131–44. 32020381 10.1007/s10928-020-09675-3 PMC7125022

[69] Boyanapalli SSS, et al. Pharmacokinetics and pharmacodynamics of Curcumin in regulating anti-inflammatory and epigenetic gene expression. Biopharm Drug Dispos. 2018;39(6):289–97. 29870054 10.1002/bdd.2136 PMC6078813

[70] Cheng D, et al. Pharmacokinetics, pharmacodynamics, and PKPD modeling of Curcumin in regulating antioxidant and epigenetic gene expression in healthy human volunteers. Mol Pharm. 2019;16(5):1881–9. 30860383 10.1021/acs.molpharmaceut.8b01246 PMC6710832

[71] Zhang C, et al. Pharmacokinetics and pharmacodynamics of the triterpenoid ursolic acid in regulating the antioxidant, Anti-inflammatory, and epigenetic gene responses in rat leukocytes. Mol Pharm. 2017;14(11):3709–17. 29035547 10.1021/acs.molpharmaceut.7b00469 PMC5697757

[72] Wu TY, et al. Pharmacokinetics and pharmacodynamics of 3,3’-diindolylmethane (DIM) in regulating gene expression of phase II drug metabolizing enzymes. J Pharmacokinet Pharmacodyn. 2015;42(4):401–8. 26138223 10.1007/s10928-015-9421-5

[73] Jia Q et al. Utilization of physiologically based Pharmacokinetic modeling in Pharmacokinetic study of natural medicine: an overview. Molecules, 2022;27(24). 10.3390/molecules27248670 PMC9782767 36557804

[74] Ferreira A, Lapa R, Vale N. PBPK modeling and simulation and therapeutic drug monitoring: possible ways for antibiotic dose adjustment. Processes. 2021;9(11):2087.

[75] Ya K, et al. Development of a physiologically based Pharmacokinetic model of mitragynine, psychoactive alkaloid in Kratom (Mitragyna speciosa Korth.), in rats and humans. J Psychoactive Drugs. 2021;53(2):127–39. 34003732 10.1080/02791072.2020.1849877

[76] Chen Y, et al. Predicting antitumor effect of deoxypodophyllotoxin in NCI-H460 tumor-bearing mice on the basis of in vitro pharmacodynamics and a physiologically based pharmacokinetic-pharmacodynamic model. Drug Metab Dispos. 2018;46(6):897–907. 29618575 10.1124/dmd.117.079830

[77] Li X, et al. Physiologically based Pharmacokinetic modelling and simulation to predict the plasma concentration profile of Schaftoside after oral administration of total flavonoids of desmodium styracifolium. Front Pharmacol. 2022;13:1073535. 36588682 10.3389/fphar.2022.1073535 PMC9794590

[78] Law FC, et al. Physiologically based Pharmacokinetic modeling of tea Catechin mixture in rats and humans. Pharmacol Res Perspect. 2017;5(3):e00305. 28603626 10.1002/prp2.305 PMC5464336

[79] Chow HS, et al. Phase I Pharmacokinetic study of tea polyphenols following single-dose administration of Epigallocatechin gallate and polyphenon E. Cancer Epidemiol Biomarkers Prev. 2001;10(1):53–8. 11205489

[80] Chow HS, et al. Pharmacokinetics and safety of green tea polyphenols after multiple-dose administration of Epigallocatechin gallate and polyphenon E in healthy individuals. Clin Cancer Res. 2003;9(9):3312–9. 12960117

[81] Lee M-J, et al. Pharmacokinetics of tea catechins after ingestion of green tea and (–)-epigallocatechin-3-gallate by humans: formation of different metabolites and individual variability. Cancer Epidemiol Biomarkers Prev. 2002;11(10):1025–32. 12376503

[82] Han D-G, et al. Impact of route-dependent phase-II gut metabolism and enterohepatic circulation on the bioavailability and systemic disposition of Resveratrol in rats and humans: A comprehensive whole body physiologically-based Pharmacokinetic modeling. Biomed Pharmacother. 2022;151:113141. 35609369 10.1016/j.biopha.2022.113141

